# Moving toward genome-editing therapies for cardiovascular diseases

**DOI:** 10.1172/JCI148555

**Published:** 2022-01-04

**Authors:** Kiran Musunuru

**Affiliations:** 1 Cardiovascular Institute,; 2 Department of Medicine, and; 3 Department of Genetics, Perelman School of Medicine at the University of Pennsylvania, Philadelphia, Pennsylvania, USA.

## Abstract

The rapid invention of genome-editing technologies over the past decade, which has already been transformative for biomedical research, has raised the tantalizing prospect of an entirely new therapeutic modality. Whereas the treatment of chronic cardiovascular diseases has heretofore entailed the use of chronic therapies that typically must be taken repeatedly and frequently for the remainder of the lifetime, genome editing will enable the development of “one-and-done” therapies with durable effects. This Review summarizes the variety of available genome-editing approaches, including nuclease editing, base editing, epigenome editing, and prime editing; illustrates how these various approaches could be implemented as novel therapies for cardiovascular diseases; and outlines a path from technology development to preclinical studies to clinical trials. Although this Review focuses on *PCSK9* as an instructive example of the various genome-editing approaches under active investigation, the lessons learned will be broadly applicable to the treatment of a variety of diseases.

## Introduction

Genome editing holds the potential to revolutionize the treatment of chronic cardiovascular diseases, such as coronary heart disease. Whereas all existing pharmacological treatments for coronary heart disease require taking pills daily or receiving injections every few weeks to months — for the rest of the lifetime, in order to accrue the full therapeutic benefit — genome editing results in permanent changes at the DNA level and offers the possibility of “one-and-done” therapies that would confer long-lasting protection against disease. Such therapies would have the dual advantages of maximizing the therapeutic effect (no missed doses) and eliminating a lack of medication adherence (an extremely common phenomenon) as an obstacle to health. The last few years have seen a wealth of promising data that point the way to a future in which many patients will benefit from genome-editing therapies. Besides outlining the varied genome-editing approaches that are now available for use, this Review focuses on the gene for which, by far, the most proof-of-concept studies have been reported — *PCSK9*, a regulator of blood cholesterol levels and driver of coronary heart disease risk — with the goal of illustrating for the reader the various ways in which the field is advancing the development of genome-editing therapies for cardiovascular diseases.

## Genome-editing approaches

Any discussion of genome-editing therapies should begin with a description of the variety of editing tools now available for use. The first generation of tools, collectively known as engineered nucleases, have two types of functionality: the ability to search for and specifically bind to a target genomic sequence, and the ability to generate a double-strand DNA break within that sequence. Newer tools have emerged from separating the two types of functionality and pairing the search-and-bind ability with any of a variety of gene-modifying activities: chemical modification of DNA bases (base editing), modification of gene expression (epigenome editing), and reverse transcription to introduce new DNA sequences copied from RNA templates (prime editing). Each of these approaches is briefly described in the following sections; a more comprehensive discussion of genome-editing tools and their research and clinical applications, along with a complete set of references (which the need for brevity precludes from including in this Review), is available elsewhere (1).

### Nuclease editing.

There are four major types of engineered nucleases: zinc-finger nucleases (ZFNs), transcription activator–like effector nucleases (TALENs), meganucleases, and clustered regularly interspaced short palindromic repeats (CRISPR)/CRISPR-associated (Cas) systems. Each has the ability to seek out specific genomic sequences and to introduce double-strand DNA breaks, although the mechanisms by which they carry out these tasks are quite different. This Review will focus primarily on the CRISPR/Cas systems, as they are the most widely used class of nucleases and have the best prospects for translation into cardiovascular therapies in the near future.

**Figure 1:**
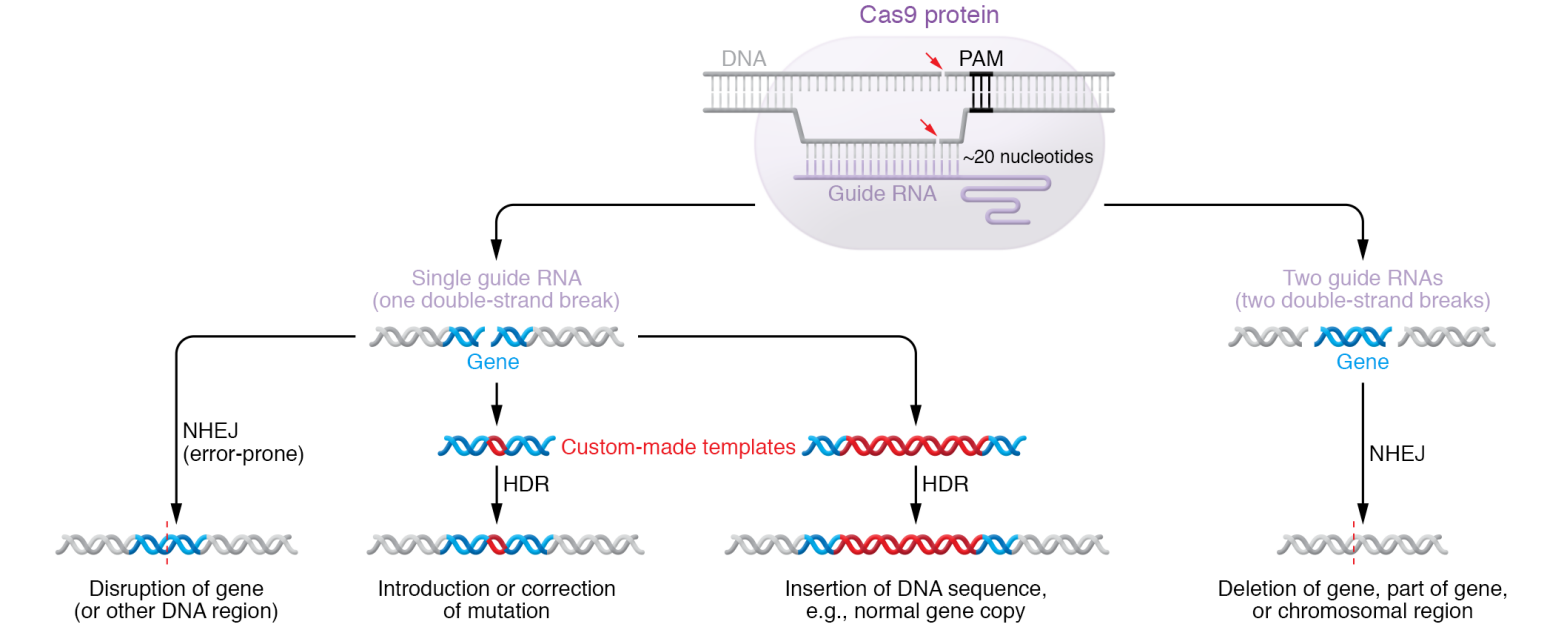
CRISPR/Cas9 nuclease editing. The protospacer-adjacent motif (PAM) in the DNA and spacer sequence in the guide RNA direct CRISPR/Cas9 to a specific genomic site. There, it generates a double-strand break (indicated by red arrows pointing to DNA strands) that is repaired by one of two repair mechanisms: non-homologous end joining (NHEJ) or homology-directed repair (HDR). In NHEJ, free DNA ends are ligated together, which can restore the original DNA sequence or introduce insertions or deletions. This strategy is suitable for disrupting genes or non-coding elements in the genome. HDR enables more precise changes but requires the addition of a template, which reduces its efficiency. Adapted with permission from the *Journal of the American College of Cardiology* (58).

The CRISPR/Cas systems of bacterial origin that are widely used for genome editing — CRISPR/Cas9 and CRISPR/Cas12 — each have two components, a Cas protein and a guide RNA (2). The guide RNA provides the search-and-bind ability, encoded within the RNA sequence itself, whereas the Cas protein has the ability to produce a double-strand break, employing either one or two cleavage domains to cut the two DNA strands. *Streptococcus pyogenes* Cas9, or SpCas9, was the first CRISPR/Cas system to be adapted for genome editing in mammalian cells (Figure 1 and refs. 3–7). Its guide RNA, about 100 nucleotides in length, encodes the DNA targeting specificity in its first 20 nucleotides, called the spacer. SpCas9 binds to the other 80 nucleotides, and the protein-RNA complex scans along any double-strand DNA molecules into which it comes in contact. As SpCas9 unwinds and scans DNA, it pauses at NGG motifs (N being any nucleotide), whereupon it will position the spacer of the guide RNA opposite the DNA strand that does not contain the NGG motif, called the target strand. If there is perfect (or, in some cases, near-perfect) complementarity of the target strand sequence and the spacer sequence, there is extensive Watson-Crick base-pairing between DNA and RNA that activates SpCas9, resulting in a double-strand break proximal to the third base pair upstream of the NGG motif. The DNA sequence on the non-target strand corresponding to the RNA spacer sequence is called the protospacer, which is the 20-nucleotide sequence just upstream of the NGG motif. The NGG motif is called the protospacer-adjacent motif (PAM).

SpCas9 has become the most popular genome-editing tool by far because of the ease of redirecting SpCas9 to any desired genomic sequence by simply changing the 20-base spacer of the guide RNA, as well as its higher rates of editing efficiency compared with other genome-editing tools (8). Nonetheless, Cas9 proteins adapted from other bacterial species are also being used for genome editing, most notably *Staphylococcus aureus* Cas9 (SaCas9), which has the dual advantages of being smaller than SpCas9 and having a different PAM sequence, NNGRRT (R is either G or A), which gives it a distinct targeting range (9). Protein engineering of SpCas9 and SaCas9 has yielded variants that recognize novel PAM sequences (10, 11). At least three other Cas proteins, all of the Cas12 family, have also proven to be effective genome editors: Cas12a/Cpf1, Cas12b/C2c1, and Cas12e/CasX (12–14).

The ultimate outcomes of nuclease editing depend on each cell’s attempt to repair the nuclease-induced double-strand break. The default repair mechanism is non-homologous end joining (NHEJ), whereby the free DNA ends are ligated together (Figure 1 and refs. 15, 16). Although the original DNA sequence is often restored, NHEJ is an error-prone process that can introduce an insertion or deletion (indel mutation) — most often one or a few base pairs, but in some cases dozens, hundreds, or even thousands of base pairs. Because the indel mutations occur in semi-random fashion, different cells will acquire different indels. Despite the unpredictability of the mutagenesis, if the goal is to simply disrupt a gene or a noncoding element — which is a strategy suitable for a variety of cardiovascular disorders, such as atherosclerotic vascular disease and transthyretin amyloidosis — NHEJ is well suited to the task, achieving up to 100% editing efficiency in some contexts. If the goal is to make a precise change with nuclease editing, such as correction of a disease-causing mutation, one must instead rely on a different cellular repair mechanism, homology-directed repair (HDR) (Figure 1 and refs. 15, 17). HDR has several substantial limitations: it requires an extra DNA repair template along with the Cas protein and the guide RNA, which complicates delivery into cells; its efficiency is typically far less than that of NHEJ, in many cases less than a few percent; and it is less active in non-proliferating cells compared with proliferating cells, making it less practical for use in key organs involved in cardiovascular diseases such as the heart and liver. Fortunately, newer genome-editing approaches such as base editing and prime editing are able to offer the precision of HDR while overcoming its shortcomings, as described below.

Nuclease editing in the context of therapeutic applications can have undesirable consequences of two kinds: unintended on-target editing, such as very large indel mutations and even chromosomal abnormalities; and off-target editing, due to the nuclease having the potential to bind to sites that are an imperfect match to the guide RNA spacer, resulting in indel mutations at sites other than the intended target site. In theory, if an off-target edit were to occur in a tumor suppressor gene or oncogene, it could confer an increased long-term risk of cancer. Alterations of either the Cas protein or the guide RNA can reduce off-target editing, though often at the cost of reduced on-target editing (18–20).

### Base editing.

Base editing (as well as epigenome editing and prime editing) takes advantage of the fact that CRISPR/Cas9 can be directed to a desired site in the genome even if the Cas9 cleavage domains are altered such that it can only cut one DNA strand (nickase Cas9, or nCas9) or neither DNA strand (dead Cas9, or dCas9). Fusion of additional domains to the nCas9 or dCas9 protein can add different types of functionality to the CRISPR/Cas9 system.

**Figure 2:**
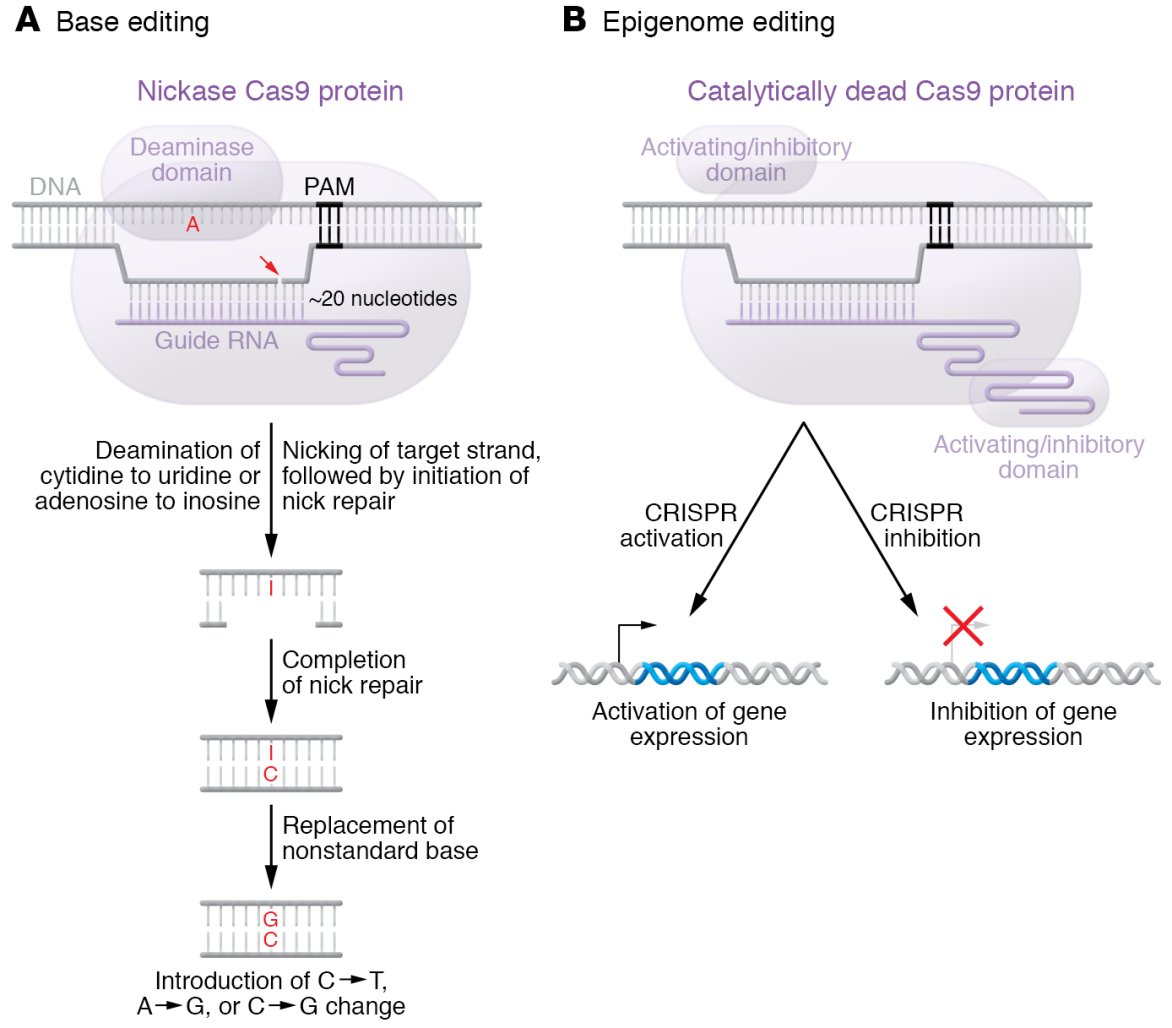
Base editing and epigenome editing. (**A**) Base-editing strategies offer the advantages of precision editing without the inefficiency that complicates the use of HDR. With base editing, only one strand is cut (or nicked), nucleotides around the site of the nick are replaced, and the nick is repaired. The specific nucleotides that undergo replacement are determined by the selection of the base editor. (**B**) In epigenome editing, catalytically dead Cas9 (dCas9) can be directed to a gene promoter or transcriptional enhancer to modify gene expression. This strategy can be used to either enhance or repress target gene expression. It does not make a change to the DNA sequence. Adapted with permission from the *Journal of the American College of Cardiology* (58).

There are two major classes of base editors, cytosine base editors that can cause C on a DNA strand to be replaced by another base (typically T, though in some cases G) (21, 22) and adenine base editors that can cause A to be replaced by G (23) (Figure 2). If nCas9 is fused to any of a variety of naturally occurring cytidine deaminase domains (e.g., from the APOBEC1 protein or the AID protein), the deaminase can potentially act upon any C within an editing window on the non-target DNA strand. (The unwinding of the DNA strands by Cas9 and hybridization of the target strand to the guide RNA create a structure known as an R-loop, which makes a portion of the non-hybridized, non-target strand into a single-strand DNA bubble that is accessible to action by the deaminase domain — thus defining the editing window, the extent of which varies depending on the specific Cas9 ortholog used.) Deamination converts C to U (uracil), which ordinarily would be restored back to C by the action of uracil-DNA glycosylase; fusion of yet another domain to nCas9, an inhibitor of uracil-DNA glycosylase, prevents this repair. nCas9 nicks only the target strand; nick repair entails removal of nucleotides around the site of the nick, followed by replacement of the nucleotides via complementarity to the non-target strand. For any U present in the non-target strand, an A goes into the complementary position in the target strand (since A and U can form a base pair). Following nick repair, the cell eventually replaces the non-standard U (normally found only in RNA) with the standard T. In this way, a C-G base pair is edited to a T-A base pair.

Because there is no naturally occurring adenosine deaminase that acts on single-strand DNA, protein evolution was used in the laboratory to create a novel DNA deaminase (23). Fusion of this evolved deaminase domain to nCas9 enables adenine base editing, which operates similarly to cytosine base editing. Within the editing window on the non-target strand, A is converted to I (inosine), nick repair occurs on the target strand, a C goes into the target strand opposite the I, and eventually the non-standard I is replaced with the standard G. Thus, an A-T base pair is edited to a G-C base pair.

Cytosine and adenine base editors are limited in the types of edits they can produce: single-nucleotide changes, largely transition mutations. But if a desired edit (e.g., introduction of a nonsense mutation to disrupt a gene, or correction of a disease mutation) is compatible with base editing, it can be achieved with very high efficiency even in non-dividing cells, in some cases approaching 100%. Off-target editing can occur, although it is typically limited to single-nucleotide changes resulting from deaminase activity.

### Epigenome editing.

If dCas9 is directed to a sequence in a gene promoter or transcriptional enhancer, it has the potential to sterically interfere with factors that normally interact with that sequence (24). Called CRISPR interference — by analogy to RNA interference mediated by short hairpin RNAs, though quite different in mechanism — this phenomenon can be exploited to knock down the expression of specific genes. CRISPR interference is more potent if dCas9 is fused to a domain, such as the KRAB (Krüppel-associated box) domain, that actively represses gene expression by modifying the local chromatin structure (Figure 2 and ref. 25). The opposite phenomenon, called CRISPR activation, is achieved either by fusing domains that enhance gene expression (such as the transcriptional activator VP16) to dCas9 or by extending the sequence of the guide RNA on its 3′ end with RNA aptamers that recruit activation domains (Figure 2 and ref. 26). With either CRISPR interference or CRISPR activation, no change is made to the DNA sequence, and the gene expression effect appears to endure only as long as the editing protein is present, i.e., the effect is transient.

A distinct type of epigenome editing involves alteration of the methylation state of DNA sequences, particularly at cytosine bases in CpG dinucleotide sequences. Methylation near the transcription start site typically is linked to gene silencing, whereas non-methylation is linked to gene activation. Fusions of methyltransferase or demethylase domains to dCas9 can decrease or increase gene expression, respectively, and the methylation changes can endure long-term while still being reversible if an epigenome editor with the opposite effect is later applied to the same genomic site (27).

### Other types of editing.

**Figure 3:**
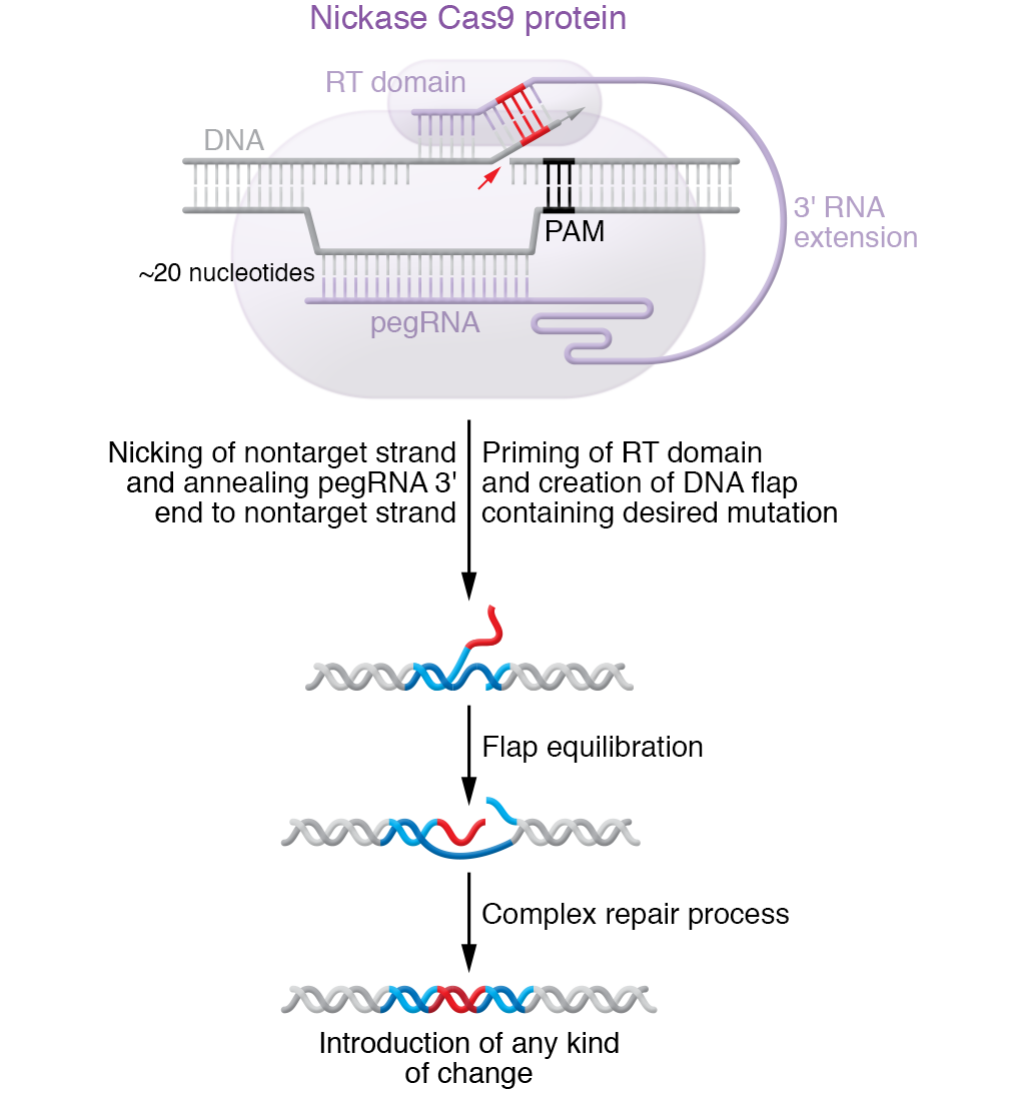
Prime editing. Prime editing overcomes limitations inherent to base editing and HDR by fusing nickase Cas9 to a reverse transcriptase (RT) that can build a DNA sequence with a desired mutation into an extended guide RNA (referred to as pegRNA). Though its efficiency tends to be lower than NHEJ or base editing, prime editing enables single-nucleotide changes of any kind as well as indel mutations of various sizes. Adapted with permission from the *Journal of the American College of Cardiology* (58).

Prime editing was recently developed with the goal of overcoming the limitations in the types of changes that can be made by base editing, as well as the limitations of HDR (28). nCas9 is fused to a reverse transcriptase that can build a DNA strand complementary to a single-strand RNA substrate. The substrate is provided by an extension of the guide RNA on its 3′ end, with an RNA sequence that is complementary to the non-target DNA strand but also includes a desired mutation. (The extended guide RNA is referred to as pegRNA.) nCas9 nicks the non-target strand, and the 3′ end of the pegRNA hybridizes with the non-target strand on one side of the nick (5′ direction), which forms an RNA-DNA duplex that serves as a template for reverse transcriptase, which in turn builds a DNA sequence with the desired mutation on the middle portion of the pegRNA (Figure 3). This new DNA strand can replace part of the non-target strand, resulting in permanent incorporation of the mutation. Although its efficiency remains lower than that of nuclease NHEJ editing or base editing (albeit higher than that of HDR editing), prime editing can precisely introduce a wide variety of mutations: single-nucleotide changes of any kind, and indel mutations of various sizes up to dozens of base pairs in length and potentially even longer. The extent of off-target editing with prime editors remains to be defined.

RNA editing is an orthogonal editing approach that uses the Cas13 family of proteins (29). Like CRISPR/Cas9 and CRISPR/Cas12, CRISPR/Cas13 systems have protein and RNA components, but they act on target RNAs rather than target DNAs. RNA editors can be used either to degrade target RNAs (29) or to make base edits (A-to-I edits or C-to-U edits) in target RNAs (30, 31). Because RNA molecules are short-lived, the persistence of the RNA effects depend on the prolonged presence of the RNA editor so that it continues to act on any newly transcribed RNA molecules.

## Therapeutic genome editing: the example of PCSK9

For cardiovascular diseases, virtually all therapeutic genome-editing applications that are under active exploration would take place within the bodies of patients, in organs like the heart and liver — in vivo applications — rather than in cells taken from the body, with the editing occurring outside the body, and then transplanted back into the body — ex vivo applications. Many of the proof-of-concept studies for in vivo therapeutic genome editing have focused on genes closely tied to a well-established, modifiable causal risk factor for atherosclerotic cardiovascular disease, namely low-density lipoprotein cholesterol (LDL-C). One gene in particular stands out: the proprotein convertase subtilisin/kexin type 9 (*PCSK9*) gene. Because this gene is preferentially expressed in the hepatocytes in the liver, and its protein product is secreted into the bloodstream, where it exerts its primary effect of increasing the blood LDL-C concentration, it has emerged as a popular target gene against which to test out new genome-editing technologies. The liver is an eminently targetable organ through a variety of delivery methods, and both the blood PCSK9 protein level and the blood LDL-C level represent easily measured pharmacodynamic markers of *PCSK9* gene editing. As such, concentrating this Review’s discussion on published *PCSK9* editing studies provides a comprehensive overview of progress in the therapeutic genome editing field and illustrates the key choices that must be made in designing genome-editing therapies, with no other genes (whether involved in cardiovascular diseases or in other diseases) having nearly the same breadth of data at the present time.

*PCSK9* is, of course, a gene of intense interest to developers of cardiovascular therapies on its own merits as a drug target. *PCSK9* was identified as a cause of familial hypercholesterolemia (genetically elevated blood LDL-C levels) via patients who were found to have single copies of gain-of-function mutations in the gene (32), whereas people with single copies of *PCSK9* nonsense (loss-of-function) mutations have substantially reduced blood LDL-C levels as well as up to 88% reduction in risk of coronary heart disease (33) without having any serious adverse health consequences (34). Furthermore, a few individuals with complete knockout of *PCSK9* via two loss-of-function mutations have been reported (35, 36). These observations have made *PCSK9* a compelling drug target for the treatment and prevention of coronary heart disease, with three PCSK9-targeting drugs already approved for use in patients — alirocumab (a monoclonal antibody that binds PCSK9 protein in the blood), evolocumab (a monoclonal antibody that binds PCSK9 protein in the blood), and inclisiran (a small interfering RNA that knocks down *PCSK9* mRNA levels in hepatocytes) — and a number of others in the drug development pipeline.

In an early demonstration of high-efficiency in vivo mammalian genome editing, an adenoviral vector, comprising a DNA core that encoded SpCas9 and a guide RNA targeting a sequence in exon 1 of the mouse *Pcsk9* gene, was used to knock down *Pcsk9* in the mouse liver by introducing loss-of-function mutations via NHEJ (37). Adenoviral vectors are generally avoided for use in patients because of the risk of severe and potentially life-threatening immune responses to the vectors; adeno-associated viral (AAV) vectors are preferred because they are better tolerated immunologically and thus more appropriate for clinical use. One disadvantage of AAV vectors is their limited cargo capacity (<5 kb of exogenous sequence) in which most genome-editing tools do not fit. For example, genetically encoded SpCas9 is about 4.2 kb, and a guide RNA expression cassette is about 500 base pairs, leaving minimal room for a promoter sequence to express SpCas9 and a polyadenylation sequence. With their much larger cargo capacity, adenoviral vectors were chosen for this initial proof-of-concept study of in vivo genome editing by CRISPR/Cas9. The investigators administered the SpCas9 vector or a control vector to wild-type mice. After several days, there was more than 50% whole-liver editing at the *PCSK9* target site; the most common edits were 1– or 2–base pair deletions or insertions, with edits as large as dozens of base pairs occurring with much less frequency. The *PCSK9* editing was accompanied by reductions of blood PCSK9 protein levels of about 90% and of blood cholesterol levels of 35%–40%, almost as much as the 36%–52% reductions of cholesterol observed in germline *Pcsk9-*knockout mice (38). This initial study showed no evidence of editing at a handful of candidate off-target sites.

In a subsequent study, a different group of investigators replicated the same editing results in mice treated with a similar adenoviral vector with SpCas9 and the same *Pcsk9* guide RNA (39). The investigators rigorously assessed for off-target editing in the liver via a two-stage strategy. They first screened for candidate off-target sites using a biochemical technique called CIRCLE-Seq, in which circularized mouse genomic DNA fragments were mixed with SpCas9 protein and the *Pcsk9* guide RNA in vitro, followed by next-generation sequencing to identify linearized DNA fragments. This procedure yielded a list of 182 candidate sites. In the second stage, the investigators performed PCR amplification of the candidate sites and deep next-generation sequencing of the amplicons from liver genomic DNA samples from the SpCas9-treated mice. They observed no editing at any of the sites, suggesting that it is possible to select genome-editing reagents with enough editing specificity that there is no detectable off-target mutagenesis in vivo.

Despite the encouraging results of these studies, they have no direct relevance to what might happen with *PCSK9* editing in human patients, in light of three major differences between mice and humans. First, there are substantial differences between the mouse *Pcsk9* and human *PCSK9* gene sequences, such that it is prohibitive to identify an effective guide RNA matching both species. Second, there are substantial differences between the mouse and human genomes, such that off-target profiling of the mouse genome would not be predictive of off-target editing in the human genome. Third, there are substantial physiological differences between mouse and human hepatocytes, such that gene editing outcomes could differ significantly between the two cell types.

For a more relevant assessment of efficacy and safety of a potential human *PCSK9*-editing therapy than can be achieved in wild-type mice, a study was undertaken in chimeric liver-humanized mice, a model system in which the endogenous mouse hepatocytes are replaced with transplanted human hepatocytes (40). Liver-humanized mice were treated with an adenoviral vector encoding SpCas9 and a guide RNA targeting a sequence in exon 1 of the human *PCSK9* gene. There was about 50% NHEJ-mediated editing of the human *PCSK9* alleles present in the humanized liver, with no editing observed at a handful of candidate off-target sites, along with about 50% reduction of human PCSK9 protein levels in the blood.

The next set of studies pivoted away from adenoviral vectors to delivery approaches more amenable to clinical use. In the first study to use AAV to achieve high-efficiency in vivo mammalian genome editing, SaCas9 along with either of two guide RNAs targeting sequences in the mouse *Pcsk9* gene was encoded in a single AAV vector (9). Either AAV vector, upon administration to wild-type mice, achieved 40%–50% whole-liver NHEJ-mediated *Pcsk9* editing, with reductions of blood PCSK9 protein levels of more than 90% and of blood cholesterol levels of about 40%, very similar to the effects observed in the aforementioned studies with adenoviral delivery of SpCas9.

The successful use of AAV was followed by the use of nonviral methods to deliver CRISPR/Cas9 into the liver in vivo. In the first study with CRISPR/Cas9 delivered into the liver solely via a nonviral method, lipid nanoparticles (LNPs) formulated either with the SpCas9 mRNA or with a synthesized guide RNA targeting a sequence in *Pcsk9* were serially injected into wild-type mice, resulting in reductions of PCSK9 protein levels within the liver by 40%–50% (41). In a subsequent study, LNPs formulated either with the SpCas9 mRNA or with a mix of two synthesized guide RNAs targeting distinct sequences in *Pcsk9* — with chemical modifications intended to enhance RNA stability in vivo — were coadministered to wild-type mice (42). LNP treatment yielded more than 80% whole-liver editing of the gene, with the very high editing rate due to extremely efficient NHEJ-mediated deletion between the sites targeted by the two guide RNAs (Figure 1). The editing resulted in absence of detectable blood PCSK9 protein and 35%–40% reductions of blood cholesterol levels. There was no editing in the lungs or spleen, implying that either the LNPs specifically targeted the liver, or the *Pcsk9* locus was accessible to SpCas9 action only in liver cells.

The next set of studies exploited the development of newer types of genome editing, ranging from base editing to epigenomic editing to RNA targeting. Cytosine base editors can directly introduce nonsense mutations into genes via C-to-T changes or G-to-A changes (the latter resulting from C-to-T edits on the antisense strand) in specific codons. An adenoviral vector encoding the cytosine base editor BE3 along with a guide RNA targeting the *Pcsk9* tryptophan-159 codon (TGG, for which G-to-A edits of either or both Gs result in a stop codon) was administered to wild-type mice (43). The treatment resulted in about 30% of the *Pcsk9* alleles in the liver being edited, mostly into the desired stop codons but with some bystander edits resulting in missense mutations, as well as indel mutations at a rate of 1%–2%. There were corresponding reductions of blood PCSK9 protein levels of about 60% and of blood cholesterol levels of about 30%.

The same *Pcsk9* base-editing strategy was used in one of the first demonstrations of fetal genome editing in mice. By analogy to fetal surgery, in which patients with life-threatening anatomical defects are treated while still in the womb, fetal genome editing would be reserved for patients with severe genetic disorders causing damage at the prenatal stage and resulting in high morbidity and mortality after birth. An adenoviral vector expressing the BE3 base editor targeting *Pcsk9* was administered to the livers of fetal mice via injection into the vitelline vein, the precursor to the portal vein (44). This prenatal procedure resulted in permanently reduced postnatal blood PCSK9 and cholesterol levels. (Please note that hypercholesterolemia is not a condition that under any circumstances would require prenatal treatment, and the mouse study was performed only as a proof of concept of fetal genome editing. The same study also reported base editing of the *Hpd* gene, resulting in the successful treatment of hereditary tyrosinemia type 1 in fetal mice.)

In a demonstration of epigenome editing, catalytically dead SaCas9 was fused to a KRAB repressor domain; the editor and a guide RNA targeting a sequence in the *Pcsk9* promoter were encoded in two separate AAV vectors (45). The AAV vectors were coadministered to wild-type mice, resulting in about 50% reductions in hepatic *Pcsk9* gene expression and about 80% reductions in blood PCSK9 protein levels, along with corresponding reductions in blood LDL-C levels. Notably, the therapeutic effects weakened over the course of a few months, suggesting that as expression of the epigenome editor waned, so too did the repression of *Pcsk9*. Another study demonstrated the use of an RNA editor, CasRx (Cas13d), delivered by an AAV vector to knock down *Pcsk9* expression (46). As with epigenome editing, the therapeutic effect would be expected to last only as long as the expression of the editor persisted. Both epigenome editing and RNA targeting would likely require repeated administrations of the treatment in order to maintain chronic therapeutic effects, unlike the “one-and-done” effects that are possible with nuclease editing or base editing.

The most recent set of studies have addressed a key step in the translation of therapeutic genome editing to human patients: demonstration of efficacy and safety in nonhuman primates. The first study used meganucleases rather than a CRISPR editor to target the *PCSK9* gene (47, 48). The investigators used an AAV vector encoding a meganuclease specific for a sequence in exon 7 of *PCSK9* and expressed from a strong liver-specific promoter. When administered to rhesus macaques via intravenous injection at various doses, the highest dose of the AAV vector resulted in 46% whole-liver *PCSK9* editing, with corresponding reductions of blood PCSK9 protein levels of 85% and of blood LDL cholesterol levels of 56%. (Lower AAV doses produced substantially lower editing rates.) The reductions persisted for at least 3 years (48).

Despite the success of the meganuclease nonhuman primate study, several aspects of the study that are relevant to clinical translation are noteworthy. First, there was unintended on-target editing. Although the goal was to disrupt the *PCSK9* gene via NHEJ, which would result in small indel mutations, in fact the most frequent editing event was integration of AAV vector sequences into the genome at the site of the double-strand break in the *PCSK9* gene, with unclear safety consequences. Second, meganuclease treatment resulted in significant off-target mutagenesis at numerous genomic sites both in the monkey livers in vivo and in human hepatocytes in vitro. Third, there were substantial T cell immune responses against both the AAV vector and the meganuclease, resulting in moderate rises in blood transaminase levels in all treated monkeys several weeks after treatment, consistent with immune-mediated hepatocyte death. The rises spontaneously resolved over the course of several weeks to months without any apparent long-term health consequences or attenuation of the *PCSK9* editing or of the blood PCSK9 and LDL-C reductions. These issues notwithstanding, this study established the feasibility of “one-and-done” genome editing in primates, with therapeutic effects lasting for several years so far and, likely, for the lifetimes of the treated animals.

In two recent nonhuman primate studies, the two teams of investigators used adenine base editing to knock down *PCSK9* in cynomolgus monkeys (49, 50). LNPs encapsulating both an adenine base editor mRNA and a synthetic guide RNA targeting the splice donor at the end of *PCSK9* exon 1 were used to deliver the editor into the liver. Whereas one study demonstrated relatively modest effects — 26% whole-liver *PCSK9* editing, 32% reductions of blood PCSK9 levels, and 14% reductions of blood LDL-C levels at 1 month after treatment (49) — the other study demonstrated larger pharmacodynamic effects more relevant to clinical translation — 66% whole-liver *PCSK9* editing, about 90% reductions of blood PCSK9 levels, and about 60% reductions of blood LDL-C levels persisting more than 8 months in an ongoing study (50). These studies contrasted with the aforementioned meganuclease nonhuman primate study in several ways. Since the LNP approach did not use any DNA components, there was no risk of vector sequence integration into the genome, and the use of base editing resulted in a specific base pair change in *PCSK9*, in contrast to the semi-random indels and AAV vector sequence insertions induced by the meganuclease. With base editing, there was no discernible off-target editing at any of a large number of candidate sites in human hepatocytes, and low-level off-target editing at just a single candidate site in monkey liver, with the off-target editing confined to single–base pair changes (rather than indels). Finally, the LNP treatment resulted in immediate, transient rises in blood transaminase levels that spontaneously resolved in 1 to 2 weeks, with no subsequent transaminitis to suggest a robust immune response — although, notably, repeated administration of LNPs to some of the animals in one of the studies did elicit the development of antibodies against the base editor.

## Prospects for translation in the near future

On the strength of all of the aforementioned studies, *PCSK9* editing appears to be poised to enter clinical trials for patients with hypercholesterolemia and coronary heart disease. The therapeutic potential for genome editing in cardiovascular diseases and other diseases, of course, extends beyond *PCSK9* (Table 1). Preclinical studies have established the prospects for the treatment of homozygous familial hypercholesterolemia by targeting the *ANGPTL3* gene in the liver (51). Although not exclusively cardiovascular diseases, accelerated mortality in patients with Duchenne muscular dystrophy or with Hutchinson-Gilford progeria syndrome is driven by cardiovascular complications arising from the heart or from the vasculature, and preclinical studies have demonstrated amelioration of cardiovascular phenotypes with Cas9 nuclease editing and base editing (52–55).

Most promisingly, a phase I clinical trial in which genome editing is being used to treat transthyretin amyloidosis with polyneuropathy by targeting the *TTR* gene (encoding transthyretin, a thyroxine and retinol transport protein) in the liver (56) is already under way. Interim results with single doses of LNPs encapsulating both Cas9 mRNA and a synthetic guide RNA targeting *TTR*, administered to six patients, demonstrated successful knockdown of blood transthyretin levels by as much as 96% at 1 month after treatment (57). Although the amount of *TTR* editing and the durability of the therapeutic effect remain to be established, and this clinical trial is focused on the treatment of polyneuropathy, the high degree of transthyretin reduction observed would be predicted to benefit patients suffering from cardiomyopathy caused by transthyretin amyloidosis.

It is likely that clinical trials with genome-editing therapies for other cardiovascular diseases will commence in the near future. Remarkably, almost all of the work forming the basis of these clinical trials has unfolded in just the last decade, and we can undoubtedly expect the next decade to see just as extraordinary a rate of progress of development of genome-editing approaches, to the ultimate benefit of patients everywhere.

## Figures and Tables

**Table 1:**
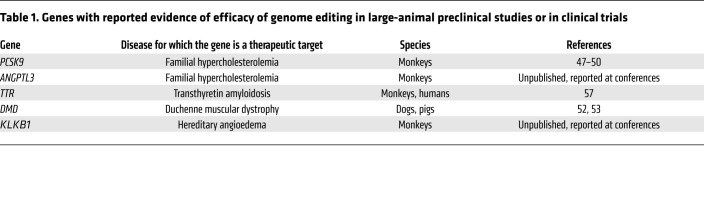
Genes with reported evidence of efficacy of genome editing in large-animal preclinical studies or in clinical trials

## Version 1 01/04/2022

Electronic publication
